# Deep learning predicts patients outcome and mutations from digitized histology slides in gastrointestinal stromal tumor

**DOI:** 10.1038/s41698-023-00421-9

**Published:** 2023-07-24

**Authors:** Yu Fu, Marie Karanian, Raul Perret, Axel Camara, François Le Loarer, Myriam Jean-Denis, Isabelle Hostein, Audrey Michot, Françoise Ducimetiere, Antoine Giraud, Jean-Baptiste Courreges, Kevin Courtet, Yech’an Laizet, Etienne Bendjebbar, Jean Ogier Du Terrail, Benoit Schmauch, Charles Maussion, Jean-Yves Blay, Antoine Italiano, Jean-Michel Coindre

**Affiliations:** 1 Owkin, Inc., New York, NY USA; 2 Cancer Research Center of Lyon, Centre Léon Bérard, Lyon, France; 3 Department of Biopathology, Institut Bergonié, Bordeaux, France; 4 Faculty of Medicine, University of Bordeaux, Bordeaux, France; 5 Department of Surgical Oncology, Institut Bergonié, Bordeaux, France; 6 Clinical Research and Clinical Epidemiology Unit, Institut Bergonié, Bordeaux, France; 7 Department of Medicine, Institut Bergonié, Bordeaux, France

**Keywords:** Sarcoma, Translational research

## Abstract

Risk assessment of gastrointestinal stromal tumor (GIST) according to the AFIP/Miettinen classification and mutational profiling are major tools for patient management. However, the AFIP/Miettinen classification depends heavily on mitotic counts, which is laborious and sometimes inconsistent between pathologists. It has also been shown to be imperfect in stratifying patients. Molecular testing is costly and time-consuming, therefore, not systematically performed in all countries. New methods to improve risk and molecular predictions are hence crucial to improve the tailoring of adjuvant therapy. We have built deep learning (DL) models on digitized HES-stained whole slide images (WSI) to predict patients’ outcome and mutations. Models were trained with a cohort of 1233 GIST and validated on an independent cohort of 286 GIST. DL models yielded comparable results to the Miettinen classification for relapse-free-survival prediction in localized GIST without adjuvant Imatinib (C-index=0.83 in cross-validation and 0.72 for independent testing). DL splitted Miettinen intermediate risk GIST into high/low-risk groups (*p* value = 0.002 in the training set and *p* value = 0.29 in the testing set). DL models achieved an area under the receiver operating characteristic curve (AUC) of 0.81, 0.91, and 0.71 for predicting mutations in *KIT*, *PDGFRA* and wild type, respectively, in cross-validation and 0.76, 0.90, and 0.55 in independent testing. Notably, PDGFRA exon18 D842V mutation, which is resistant to Imatinib, was predicted with an AUC of 0.87 and 0.90 in cross-validation and independent testing, respectively. Additionally, novel histological criteria predictive of patients’ outcome and mutations were identified by reviewing the tiles selected by the models. As a proof of concept, our study showed the possibility of implementing DL with digitized WSI and may represent a reproducible way to improve tailoring therapy and precision medicine for patients with GIST.

## Introduction

Gastrointestinal stromal tumor (GIST) is the most common mesenchymal neoplasm of the gastrointestinal tract, and is characterized by a variable clinical behavior. GIST arises from the interstitial cells of Cajal^1^ and most cases have activating mutations in the tyrosine kinase coding genes *KIT* or platelet-derived growth factor receptor alpha (*PDGFRA)*, which results in oncogenic addiction^2,3^. Imatinib, a tyrosine kinase inhibitor (TKI) targeting mutated activated *KIT* and *PDGFRA*, has been a breakthrough in the treatment of advanced phases of the disease^4,5^. Notably, response to imatinib treatment is dependent on the type of mutation. Patients with *KIT* exon 11 and exon 9 mutations respond well to imatinib at different dosages (double dose may be needed for patients with mutations in *KIT* exon 9)^6^ while patients with tumors harboring *PDGFRA* exon 18 D842V mutations are resistant^7^. The standard management of a localized GIST is complete surgical resection^8^, but about 20 to 40% of patients relapse with mainly secondary peritoneal and/or liver locations^9^. Adjuvant therapy with imatinib has been proved to benefit patients with a high risk of relapse^10^. Therefore, it is crucial to accurately identify this group of patients at initial diagnosis. The most used system for evaluating relapse risk of GIST is the Armed Forces Institute of Pathology (AFIP)/Miettinen classification (Miettinen) based on tumor location, tumor size and mitotic count per 5 mm^[211^. Mitotic count is an essential factor of this classification, but reproducibility between pathologists is not perfect^12^. In a retrospective study (see Methods for details), the crude proportion of agreement observed between the outside laboratories and the reference center laboratories was 88.9% for the mitotic count (<=5 or >5 mitoses/5 mm^2^) with a discordance of 6.3% in the resulting Miettinen classification. Moreover, the mitotic count is not always possible in small samples (i.e., core needle biopsies). Deep learning on virtual slides has been successfully used for the classification of tumors^13^ and prediction of survival^14^ and molecular abnormalities^15^. In this study, we evaluated the efficacy of deep learning models for predicting both patient prognosis and mutational profile of GIST on digitized hematoxylin, eosin and saffron (HES)-stained whole slide images (WSI).

## Results

### Deep learning and prediction of patients’ outcome

**Fig. 1:**
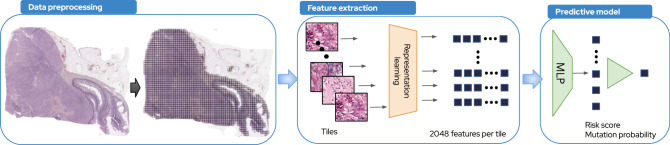
Deep learning workflow. Our DL pipeline consists of three main steps. Data preprocessing. For one raw WSI, we apply a matter detection algorithm on it in order to extract the tissue area and to remove the artifacts (blur area, etc). On the extracted tissue area, we apply a tiling which consists of dividing the whole-slide images into tiles of 112 × 112 μm (224 × 224 pixels) at a zoom level of 0.5 microns per pixel. Feature extraction. We trained from scratch a 50-layer ResNet using an inhouse dataset of sarcoma of 1287 WSI (942,626 tiles) and Momentum Contrast v2 (MoCo v2) algorithm, a self-supervised learning algorithm based on contrastive loss. Using this model, we extracted 2048 features from each of the tiles, such that a slide could be represented as a *N* × 2048 matrix with N equals to the total number of tiles. Predictive models. MLP were trained using the 2048 features to predict mutation types or survival risk.

We investigated the prognostic power of DL (the full DL workflow is shown in Fig. 1) in three subgroups of patients using different endpoints: recurrence-free survival (RFS) for patients with localized GIST and without adjuvant therapy (*N* = 161), RFS for patients with localized GIST and treated with adjuvant therapy (*N* = 66) and overall survival for patients with advanced GIST and treated with imatinib (*N* = 131, 69 metastatic at diagnosis and 62 recurrent, Supplementary Fig. 1). All models were trained using cross-validation with the whole cohort and evaluated within these subgroups (Methods) using data from center 1 and tested independently using the data from center 2.

**Fig. 2:**
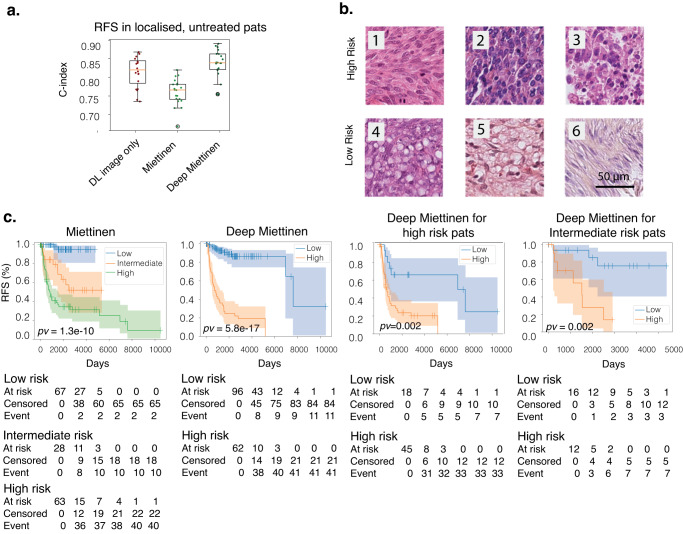
DL predicts risk of relapse from HES slides. **a** Concordance index of predicting incidents using HES slides alone (dark red), Miettinen risk criteria(light green) and Deep Miettinen(dark green) for RFS in localized, untreated patients. Shown is the distribution across 5 repetitions of 4-fold cross-validation (n = 20 for each boxplot). All boxplots demarcate quartiles and median values, while whiskers extend to 1.5× the interquartile range. **b** Example of tiles with high and low (indicated on the left) estimated risk based on the histopathological features. The most predictive tiles for high risk of relapse showed mitoses (1), high cellular density (2) and necrosis (3) while tiles with low risk of relapse showed cytoplasmic vacuolization (4, 5) low cellular density (6). **c** Kaplan–Meier plots shown for RFS for localized untreated patients within different categories. From left to right: risk groups defined by Miettinen; risk groups defined by Deep Miettinen (Low risk corresponds to risk score <mean + 0.2*std); risk groups defined by Deep Miettinen for high risk patients defined by Miettinen; risk groups defined by Deep Miettinen for intermediate risk patients defined by Miettinen.

DL showed an improvement in predicting RFS from images alone for patients with localized GIST and without adjuvant therapy (C-index = 0.81, std = 0.04 from CV in center 1) compared to the Miettinen (C-index = 0.76, std = 0.04). The performance of the multimodal DL model that includes HES images, tumor location and tumor size (Deep Miettinen) is numerically higher but was not significant compared to the model using images alone (C-index = 0.83, std = 0.04, Fig. 2a). We obtained a C-index of 0.72 (95% CI = [0.63, 0.80]) for localized untreated patients in center 2 using the Deep Miettinen model. Image only model achieved a C-index of 0.62(95% CI = [0.58, 0.66]) and the Miettinen model achieved a C-index of 0.73(95% CI = [0.62, 0.82]). No difference in performance between Deep Miettien and Miettinen models was observed in center 2.

In terms of risk stratification, the difference of the estimated relapse risk between high and low-risk groups defined by the Deep Miettinen was greater than that defined by the Miettinen model. (chi-square statistic = 70.05, log-rank test p-value = 58e-17 and chi-square statistic = 45.56, log-rank test *p* value = 1.3e-10 for Deep Miettinen and Miettinen respectively for localized untreated patients in the training set). These differences are shown in the Kaplan-Meier curve in Fig. 2c.

Furthermore, the Deep Miettinen model was capable of stratifying classical Miettinen intermediate and high-risk groups into subgroups of different prognoses (log-rank test *p* value = 0.002 for both intermediate and high-risk groups, Fig. 2c). Of note, when testing the Deep Miettinen model in center 2, none of the patients was labeled as high-risk using the threshold estimated from center 1. Therefore, a dataset-adapted threshold for high and low-risk groups was used (a cutoff that led to a comparable number of high-risk patients as defined by the Miettinen). A higher median RFS time was observed in the low-risk group compared to the high-risk group estimated by Deep Miettinen for both intermediate and high-risk groups defined by Miettinen. However, the tests did not meet statistical significance (log-rank test *p* value = 0.29 and 0.31 for intermediate and high-risk groups respectively, Supplementary Fig. 2).

When examining the predictive tiles that were related to different risks, we noticed that mitoses, high cellular density and necrosis were associated with high-risk while cytoplasmic vacuolization and low cellular density were associated with low-risk (Fig. 2b, Supplementary Table 1–3).

For localized and treated GIST, we obtained C-index = 0.81, std = 0.1 for DL with image only, C-index = 0.68, std = 0.11 for Miettinen and C-index = 0.84, std = 0.1 for Deep Miettinen; however, these results were not validated in center 2 (C-index = 0.44 for DL with image only, C-index = 0.54 for Miettinen and C-index = 0.45 for Deep Miettinen).

For advanced GIST receiving imatinib, the performance was poor in both center 1 and center 2 (C-index = 0.52, std = 0.1 for DL with image only, C-index = 0.46, std = 0.1 for Miettinen and C-index = 0.64, std = 0.15 for Deep Miettinen in center 1 and C-index = 0.64 for DL with image only, C-index = 0.49 for Miettinen and C-index = 0.62 for Deep Miettinen in center 2).

### Deep learning and prediction of mutations

The mutational profile is important for the treatment decision and the outcome of GIST patients. We investigated the predictive power of DL on WSI for mutation classification. The main mutations were in *KIT* exon 11 (Center 1: 61.5% and Center 2: 58.4%), *PDGFRA* exon 18 (Center 1: 14.0% and Center 2: 13.4%), and *KIT* exon 9 (Center 1: 8.2% and Center 2: 7.1%; Tables 1 and 2).

**Table 1:** Center 1 cohort description.

Patient characteristics of cohorts from center 1		
	Mutation prediction	Recurrence prediction
Gender, n (%)	*N* = 1233	*N* = 305
Male	661 (54%)	180 (59.0%)
Female	572 (46%)	125 (41.0%)
Age, years		
Median	66	64
Range	19–97	19–97
Type of sampling		
Tumor resection	1002 (81.3%)	244 (80.0%)
Open biopsy	42 (3.2%)	10 (3.3%)
Core needle biopsy	191 (15.5%)	51 (16,7%)
Tumor site		
Gastric	677 (54.9%)	167 (54.8%)
Small intestine	357 (29.0%)	97 (31.8%)
Colon or rectum	53 (4.3%)	13 (4.3%)
NA	14 (1.1%)	4 (1,3%)
Others	132 (10.7%)	24 (7.8%)
Type of tumor cell		
Spindle	716 (58.0%)	
Epithelioid	351 (28.5%)	
Mixt	166 (13.5%)	
Mutated exon		
KIT exon 11	758 (61.5%)	180 (59.0%)
KIT exon 9	101 (8.2%)	30 (9.8%)
PDGFRA exon 18	173 (14.0%)	35 (11.5%)
Wild type	130 (10.5%)	38 (12.5%)
Others	71 (5.8%)	14 (4.6%)
Tumor size		
<5 cm		70 (23.0%)
5–10 cm		126 ((41.3%)
>10 cm		103 ((33.8%)
NA		6 (.,9%)
Mitotic count, per 5 mm^2^		
<5		133 (43.6%)
5–10		62 (20.3%)
>10		100 (32.8%)
NA		10 (3.3%)
Risk Group (AFIP)		
Low risk		74 (24.3%)
Intermediate risk		54 (17.7%)
High risk		170 (55.7%)
NA		7 (2.3%)

**Table 2:** Center 2 cohort description.

Patient characteristics of cohorts from center 2		
	Mutation prediction	Recurrence prediction
Gender, n (%)	N = 238	N = 286
Male	113 (47.5%)	143 (50.0%)
Female	125 (52.5%)	143 (50.0%)
Age, years		
Median	64	64
Range	20-91	20-91
Type of sampling		
Tumor resection	169 (71.0%)	207 (72.4%)
Open biopsy	23 (9.7%)	25 (8.6%)
Core needle biopsy	46 (19.3%)	54 (18.0%)
Tumor site		
Gastric	127 (53.4%)	148 (51.8%)
Small intestine	55 (23.1%)	73 (25.5%)
Colon or rectum	17 (7.1%)	23 (8.0%)
Others	39 (16.4%)	42 (14.7%)
Type of tumor cell		
Spindle	170(74.5%)	220(76.9%)
Epithelioid	41(17.8%)	46(16.1%)
Mixt	18(7.8%)	20(7.0%)
Mutated exon		
KIT exon 11	139 (58.4%)	139 (48.6%)
KIT exon 9	17 (7.1%)	17 (5.9%)
PDGFRA exon 18	32 (13.5%)	32 (11.2%)
Wild type	30 (12.6%)	30 (10.5%)
Others	20 (8.4%)	20 (7.0%)
NA		48 (16.8%)
Tumor size		
<5 cm		73 (25.6%)
5–10 cm		119 (41.7%)
>10 cm		89 (31.1%)
NA		5(1.6%)
Mitotic count, per 5 mm^2^		
<5		139 (48.6%)
5–10		61 (21.3%)
>10		77 (26.9%)
NA		9 (3.2%)
Risk Group (AFIP)		
Low risk		86 (301%)
Intermediate risk		51 (17.8%)
High risk		130 (45.4%)
NA		19 (6.6%)

**Fig. 3:**
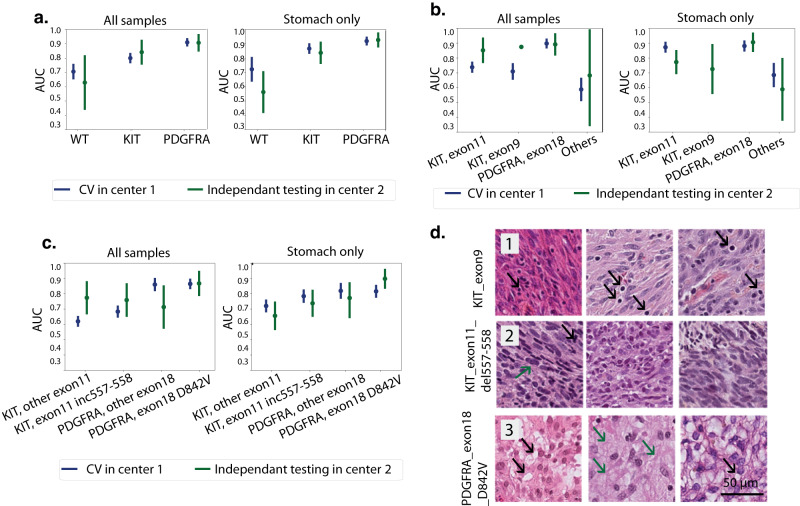
DL predicts mutations from HES slides. **a** Line plots with AUC values for mutation classification at gene level. Shown for all samples (left) and samples from stomach tumors only (right). Each point corresponds to the average AUC from 4-fold cross validation. AUCs from left-out fold during training are shown in blue and AUCs from independent testing are shown in green. Error line indicates the CI estimated using cvAUC. **b** Line plots with AUC values for mutation classification at exon level. **c** Line plots with AUC values for mutation classification at codon level. **d** Example of correctly predicted tiles for each mutation at codon level. 3 tiles per mutation are shown with the mutation type indicated on the top: the most predictive tiles for *KIT* exon9 mutation (1) showed lymphoid infiltrate (black arrows); the most predictive tiles for *KIT* exon11 del557_558 (2) showed mitoses (black arrow) and nuclear hyperchromasia (green arrow) and the most predictive tiles for *PDGFRA* exon18 D842V (3) showed vacuolization of cells (black arrows) and myxoid stroma (green arrows).

We first tested DL models in mutation classification at the gene level (*KIT* mutant, *PDGFRA* mutant or wild-type). Patients without mutations or with mutations in genes other than *KIT* and *PDGFRA* were considered Wild-type. We obtained a macro AUC of 0.81 from CV in center 1 (weighted precision = 0.75 and weighted recall=0.78, Table 3, Fig. 3a, Supplementary Fig. 3). This model was validated in center 2 with a macro-AUC of 0.74 (weighted precision = 0.68 and weighted recall = 0.78, Table 3, Fig. 3a). We obtained a better performance for tumors from the stomach in both centers (Table 3, Fig. 3a).

**Table 3:** DL models training and testing results on the mutation classification task.

	AUC[95% CI]			
	CV from training in center 1		independant testing in center 2	
	All samples	Stomach only	All samples	Stomach only
KIT	0.81[0.78, 0.84]	0.87[0.84, 0.90]	0.84[0.76, 0.91]	0.85[0.77, 0.93]
PDGFRA	0.91[0.90, 0.94]	0.92[0.90, 0.94]	0.93[0.88, 0.97]	0.92[0.86, 0.97]
WT	0.71[0.66, 0.75]	0.72[0.64, 0.80]	0.56[0.42, 0.70]	0.63[0.45, 0.82]
PDGFRA exon18	0.90[0.87, 0.92]	0.88[0.85, 0.91]	0.90[0.84, 0.96]	0.89[0.82, 0.96]
KIT exon11	0.74[0.71, 0.77]	0.87[0.84, 0.90]	0.77[0.70, 0.85]	0.85[0.77, 0.93]
KIT exon9	0.71[0.66, 0.75]	NA[NA, NA]	0.73[0.56, 0.89]	0.87[NA, NA]
Other mutations	0.60[0.53, 0.67]	0.69[0.61, 0.76]	0.59[0.39, 0.79]	0.69[0.36, 1]
PDGFRA_Exon18 D842V	0.87[0.84, 0.90]	0.82[0.78, 0.86]	0.90[0.84, 0.96]	0.87[0.79, 0.95]
PDGFRA_Exon18 other mutation	0.86[0.83, 0.90]	0.82[0.78, 0.86]	0.78[0.67, 0.88]	0.72[0.58, 0.85]
KIT_del-inc557-558	0.69[0.65, 0.72]	0.79[0.75, 0.83]	0.74[0.66, 0.82]	0.76[0.66, 0.87]
KIT_Exon11 other mutation	0.62[0.59, 0.65]	0.72[0.69, 0.76]	0.66[0.57, 0.75]	0.78[0.66, 0.88]

Next, we built predictive models for WT, *PDGFRA* exon 18, *KIT* exon 11, *KIT* exon 9 and other mutations in *KIT* or *PDGFRA*. We obtained a macro AUC of 0.76 from CV in center 1 (Weighted precision = 0.54 and weighted recall=0.60) and a macro=AUC of 0.69 (weighted precision = 0.53 and weighted recall=0.66) in center 2(Table 3, Fig. 3b, Supplementary Fig. 3). Similar to the gene-level mutation classification model, we obtained better performance for tumors from the stomach. Results are shown in Table 3 (Fig. 3b, Supplementary Fig. 3).

Finally, we investigated if DL could predict mutation types at the codon level with 2 particular types: *PDGFRA* exon18 D842V mutation, which is associated with imatinib resistance^7^ and *KIT* codons 557/558 deletion (del-inc 557/558) mutations which have been reported to be associated with a worse prognosis when compared to other *KIT* exon 11 mutations^16–18^. For the *PDGFRA* exon 18 D842V mutation, we obtained an AUC = 0.87 (95% CI = [0.84, 0.90]) in cross-validation from center 1 and AUC = 0.90 (95% CI = [0.84, 0.96]) in testing from center 2 for all samples. Comparable results were obtained for samples from the stomach only (AUC = 0.82, 95% CI= [0.78, 0.86] in center 1 and AUC = 0.87, 95%= [0.80, 0.95] in center 2, Table 3, Fig. 3c, Supplementary Fig. 3). Cutoff was selected for each mutation class to achieve a sensitivity of 90%. The model achieved a specificity of 26% using a cutoff of 0.29 for all samples and a specificity of 52% using a cutoff of 0.34 for the stomach only. For the *KIT* del-inc 557/558 mutations, we obtained an AUC = 0.69 (95% CI= [0.65, 0.72]) in center 1 and AUC = 0.76 (95% CI= [0.66, 0.86]) in center 2 for all samples and an AUC = 0.78 (95% CI= [0.75, 0.83] in center 1 and AUC = 0.74 (95% CI= [0.66, 0.82]) in center 2 for samples from the stomach only. For a sensitivity of 90%, the model achieved a specificity of 43% using a cutoff of 0.31 for all samples and a specificity of 40% using a cutoff of 0.32 for the stomach only. When reviewing the most predictive tiles for these different mutations, we observed that the *PDGFRA* exon 18 D842V mutation was associated with epithelioid or mixt cell morphology, cytoplasmic vacuolization, myxoid stroma, and lymphoid infiltrate. In contrast, *KIT* del-inc 557/558 mutation was associated with mitotic activity and nuclear hyperchromasia and *KIT* exon 9 mutation was associated with lymphoid infiltrate (Fig. 3d, Supplementary Tables 1–3).

#### Comparison of performance between DL and tumor cell morphology for mutation prediction

Next, we built mutation predictive models (baseline models for mutation classification, see Methods) based exclusively on tumor cell morphology (spindle cell, epithelioid cell or mixed). These models showed inferior performance for mutation prediction compared to DL (AUC = 0.8, 95%CI= [0.76, 0.86] for *PDGFRA* exon 18, AUC = 0.66, 95%CI= [0.60, 0.70] for *KIT* exon 11, AUC = 0.56, 95%CI= [0.49, 0.63] for KIT exon 9, AUC = 0.57, 95%CI= [0.47, 0.67] for other mutations and AUC = 0.47, 95%CI= [0.4, 0.54] for WT in center 1 and AUC = 0.71, 95%CI= [0.61, 0.81] for PDGFRA exon 18, AUC = 0.57, 95%CI= [0.52, 0.62] for KIT exon 11, AUC = 0.54, 95%CI= [0.45, 0.64] for KIT exon 9, AUC = 0.54, 95%CI= [0.38, 0.69] for other mutations and AUC = 0.58, 95%CI= [0.49, 0.68] for WT in center 2, test DeLong *p* value = 0.003 for *PDGFRA* exon18, p-value = 8.9e-05 for *KIT* exon11, *p* value = 0.004 for *KIT* exon9, *p* value = 0.09 for other mutations and *p* value = 0.02 for WT in center 1 and *p* value = 0.0004 for *PDGFRA* exon18, p-value = 0.03 for *KIT* exon11, *p* value = 0.008 for *KIT* exon9, *p* value = 0.16 for other mutations and *p* value = 0.8 for WT in center 2, Supplementary Fig. 4). Importantly, *PDGFRA* exon 18 mutations are known to be associated with epithelioid cell morphology in the stomach, while KIT exon 11 mutations are associated with spindle cell morphology. Therefore, we compared the performance of the baseline model using samples only from the stomach and obtained anAUC=0.80(95%CI= [0.71, 0.93]) for *PDGFRA* exon18 mutations and an AUC = 0.78(95%CI= [0.72, 0.82]) for *KIT* exon 11 mutations, both of which were significantly inferior to DL (test DeLong=0.001 for both *PDGFRA* exon18 and KIT exon 11).

## Discussion

In the current study, we have shown that a deep learning model can predict outcomes and mutations directly from HES-stained WSIs of GIST to a certain degree. We were particularly interested in predicting local/distant recurrence-free survival (RFS) for patients with localized GIST since the use of adjuvant therapy is known to have an impact on patients’ baseline risk stratification and prognosis^8^. In center 1, deep Miettinen model outperformed the traditional Miettinen evaluation system for predicting outcome in localized GIST with no adjuvant imatinib suggesting that DL was able to extract additional prognostic data from histological images, beyond mitotic activity. In addition, unlike the Miettinen system that includes an intermediate risk category, the Deep Miettinen model was able to stratify patients into two distinct high and low-risk groups. Moreover, the Deep Miettinen model was able to stratify further patients falling in the high and intermediate-risk categories of the conventional Miettinen system. When reviewing the predictive tiles related to high and low risks, we observed that in addition to mitoses, high cellular density, and necrosis were associated with a high risk while cytoplasmic vacuolization and low cellular density were associated with a low risk. However, the performance of our model decreased from center 1 to center 2. This is probably related to the small size of our training set as the lack of diversity in small cohorts often leads to poor transferability of deep learning models due to domain shift^19–22^. Indeed, a shift in risk prediction was observed when applying our model in the testing cohort, resulting in an under estimation of patients’ risk of relapse. This shift needs to be addressed for clinical usages. Several research directions are promising to deal with the ‘domain shift’ problem. For instance, train a feature extractor with images from multiple centers using different tissue preparation protocols, scanners, compression algorithms and compression rates to extract invariant image features from different domains. Another approach is to train a predictive model with a larger dataset or multiple datasets so that the model will generalize better in unseen data. Despite the decrease in performance across centers, our results are promising. If validated and improved using additional cohorts, they could be potentially integrated into clinical practice, particularly for predicting RFS in patients with localized GIST.

We also evaluated DL models in GIST patients treated with TKIs. DL gave good results for predicting RFS in patients with localized GIST that received adjuvant treatment in center 1, but this was not validated in center 2. Results of DL were poor in patients with advanced GIST receiving imatinib. These data suggest that the treatment effect is crucial to patients’ prognosis and models built on baseline images alone are not capable of predicting the outcome.

Since the mutational profile is essential for the treatment decision and outcome of GIST patients, we investigated the predictive power of DL on WSI for mutation classification. Our results suggest that DL models can predict mutation type at the gene, exon and codon levels with good performance. The link between histology and mutation is well in line with previous studies that showed that the mutational landscape of GIST correlates with histological features^23,24^, particularly with tumor cell morphology (spindle versus epithelioid) and tumor site. Thus a gastric tumor composed of epithelioid cells often harbors a *PDGFRA* exon 18 mutation. However, the correlation between tumor cell morphology (even associated with tumor site) and mutations was inferior to mutation prediction using DL. These results suggest that the tumor cell morphology can only partially explain the mutational profile, and DL learns additional morphological features related to mutations. With this unprecedented large cohort of patients, the model performance and robustness have been greatly improved for predicting mutations at gene level compared to a previous study^19^. More importantly, DL was able to accurately predict mutations that have a great impact in patients’ treatment decision and prognostic estimation such as *PDGFRA* exon18 D842V mutation which is a predictor for imatinib resistance and avapritinib^25^ sensitivity, and *KIT* del-inc 557/558 mutations which are associated with a worse prognosis and high-risk of relapse. While the models’ performance for predicting mutations are remarkable, it is still not perfect to replace genetic testing. However, these models can serve as pre-screening tools to greatly reduce the cost by pre-selecting a subset of patients for genetic testing, like it has been done for genotyping colorectal cancer^26^.

The black-box characteristic of deep learning is often mentioned as a drawback of such systems, especially for medical decision-making. In this study, we have shown that the systematic review of extremal tiles identified by the DL models as predictive is useful for understanding how the DL models made the predictions.

Before our system can be used in clinical practice, some limitations need to be addressed. The cohorts used for outcome prediction were relatively small, particularly when studying subgroups defined by tumorstage (localized versus advanced GIST) and treatment. Larger and heterogeneous cohorts should be used in training to develop a more robust model, which should then be validated extensively with additional cohorts. As we have seen that the Miettinien system works well and is robust, another direction of future improvement of the DL prognostic model is to integrate DL based mitotic counts into the Miettinien evaluation system to predict relapse risk. Several studies^27–30^, have shown that deep learning models could improve the accuracy and reproducibility of mitotic count. This approach could lead to more straightforward and human interpretable DL models and increase the prognostic power and reproducibility of the Miettinen evaluation system.

In conclusion, this study shows that DL could help to predict the risk of progression in localized GIST but needs improvements to be used in the clinical management of patients. DL seems more robust for identifying somatic mutations directly from HES whole slide images of patients with GIST. Such systematic tools could be useful for the management of GIST patients, especially those with localized disease. The DL method could be helpful to speed up therapeutic decisions by predicting *PDGFRA* exon18 D842V mutation in intermediate- risk Miettinen patients, which generally do not need adjuvant treatment and in high-risk Miettinen patients who need to receive avapratinib treatment^25^. This method could benefit countries where molecular techniques are not readily available. Moreover, DL could also be used as a potential research tool for discovering novel predictive histological features from whole slide images.

## Methods

### Description of patient cohorts

Two cohorts of patients with GIST were extracted from the Sarcoma Clinical and Biological database (https://sarcomabcb.org/), a data warehouse of clinical, pathological and molecular data^31^. The first cohort from Bergonié Institute (France) served as a training dataset (center 1). It consisted of 1233 patients with a GIST with mutation status. For 305 patients, treatment and follow-up are known (Table 1). These patients were mainly from south west of France. The second cohort from Center Léon Bérard (France) was used as an independent testing set with 286 patients with information on treatment and follow-up and 238 patients with mutation status (center 2). These patients were mainly from south east of France (Table 2). Eligible patients were required to have a tumor located in the gastrointestinal tract with a tumor morphology compatible with GIST and positive immunostaining for the KIT and/or the discovered on GIST-1 (DOG1) proteins. The following data were collected: gender, age at diagnosis, tumor site, type of sampling (resection, open biopsy, core needle biopsy), type of tumor cells (spindle, epithelioid, mixt). For patients with treatment and follow-up knowledge, additional data were collected: tumor size, mitotic count per 5 mm^2^, risk group according to the AFIP criteria (Tables 1 and 2), surgery (yes/no), date, type and result of surgery, targeted therapy (yes/no), date and type of targeted therapy, recurrence of disease, date and site of recurrence, last news date and status, death (yes/no), date and cause of death. All cases are recorded in the database of the French Sarcoma BCB (https://sarcomabcb.org/), which is approved by the National Committee for Protection of Personal Data (Commission Nationale de l’Informatique et des Libertés - CNIL, no. 910390). The study was declared to the CNIL on the 3rd April 2020 under the Sar-IA-Path project number MR 0012030420 and an informed consent form with nonopposition principle for the reuse of health data for research purposes was communicated to the patients of this study. The study was approved by the Institutional Review Board of both centers (Collège de Recherche Clinique for the Institut Bergonié and Comité de Revue des Études Cliniques for the Centre Léon Bérard.). Written informed consent was obtained from all patients.

### Sequencing

Mutational analysis of *KIT* and *PDGFRA* was performed based on DNA isolated from formalin-fixed, paraffin-embedded. Mutation of *KIT* exons 9, 11, 13, and 17 and *PDGFRA* exons 12, 14, and 18 was identified by Sanger sequencing of PCR products (Tables 1 and 2). Patients with no mutation in these 7 exons were considered as wild type (WT) GIST.

### Whole slide digitization

For each tumor, one representative formalin-fixed, paraffin-embedded, HES-stained slide was digitized at 40X magnification using a Hamamatsu Nanozoomer Series scanner (Hamamatsu Photonics, Hamamatsu, Japan) in Center 1 and an Aperio AT2 (Leica Biosystems, France) in Center 2. *Tissue segmentation*. We trained an Unet with a VGG11 encoder pre-trained with ImageNet^32^ and a decoder trained from scratch to segment the tissue sections from the whole slide images. The Unet was trained with an annotated dataset collected from publicly available sources containing 570 whole slides images of hematoxylin and eosin (H&E), HES and immunohistochemistry staining. Tissue areas were annotated by a pathologist inhouse for all slides.The model was trained in 460 H&E and IHC slides and validated in 115 slides. The model was trained on patches of size 2048 × 2048 μm (512 × 512 px) extracted from the WSI during 400 epochs, with a batch size of 2 slides using data augmentation such as center crop, color jitter (brightness, contrast, saturation, hue) and a standardization. A learning rate of 0.0003 has been used for the training The tissue segmentation model achieved a dice score of 0.96. By applying this algorithm, we removed all regions without tissue or artifacts such as pen marks on the slides. *Tiling*. The application of deep-learning algorithms to histological data is a challenging problem, particularly due to the high dimensionality of the data (up to 100,000 × 100,000 pixels for a single whole-slide image) and the small size of available datasets. We divided the whole-slide images into tiles of 112 × 112 μm (224 × 224 pixels) at a zoom level of 0.5 microns per pixel. *Feature extraction*. In order to extract imaging features from the tiles, we trained from scratch a 50-layer ResNet^33^ using an inhouse dataset of sarcoma of 1287 WSI from 1261 Soft Tissue Sarcoma patients (equally distributed between Female and male) diagnosed with complex genomic profiles from Institut Bergonié. The dataset contains both primary site tumor and metastases. All WSI were digitized using a Hamamatsu Nanozoomer Series scanner (Hamamatsu Photonics, Hamamatsu, Japan) at magnification of 40X. The model was trained with 942,626 tiles at a zoom level of 0.5 microns per pixel for 60 epochs with a batch size of 768 using LARS optimizer (learning rate of 0.3, momentum of 0.9, weight decay of 1.5e-6 and eta of 1e-3) and Momentum Contrast v2 (MoCo v2) algorithm^34^, a self-supervised learning algorithm using contrastive loss to shape one embedding space where different augmented views of the same image are close together. Heavy data augmentation was used during the training process in order to extract robust features that are invariant to rotation, color changes and blur (random rotation of 90°, 170° and 280°; random vertical and horizontal flip; random crop with scale = (0.2 to 1); random grayscale; random color jitter with brightness=0.8, contrast=0.8, saturation=0.8, hue=0.2; gaussian blur with sigma = (0.1, 2.0)). Using this model, we extracted 2048 features from each of the tiles, such that a slide could be represented as a N × 2048 matrix with N equals to the total number of tiles. *Blur filtering*. Additional filter based on the weighted gradient magnitude (using Sobel operator) was applied to remove blurred tiles (tiles with a weighted gradient magnitude smaller than 15 for more than half of the pixels). The tiles (RGB 0-255) were first converted to grayscale using the function of cvtColor from the openCV library. Vertical and horizontal sobel derivatives were calculated using the Sobel function from openCV with a kernel size of 3. Weighted gradient magnitudes were calculated using weight = 0.5 from both directions. *Tumor segmentation*. We trained a MLP model (with an input layer of 2048 neurons, one hidden layer of 256 neurons and one fully connected layer) to segment the tumor regions from the WSI. The model was trained at the tile level using 1259 annotated whole slides by an expert soft-tissue pathologist (JMC) in center 1 (50 randomly selected tiles from each WSI were included in the training, loss function=BCEloss, optimiser=Adam, lr=3e-3, batch size=32, epochs=10). All models were trained using GPU.

### Weakly supervised learning

Weakly supervised learning exploits the global labels (WSI-level) annotations to automatically infer local-level (pixel/patch-level) information. This paradigm is particularly well suited to our problem where the global-level information is available in the form of image level labels, e.g., mutated or wild type, but pixel-level annotations are more difficult to obtain. For both classification and prognosis models, we used a multilayer perceptron (MLP) model with an input layer with 2048 neurons, 2 hidden layers with 512 neurons and one fully connected layer with 0.25 dropout rate^35^ . Models were trained with learning rate = 3e-4, batch size = 32, optimiser=Adam, loss function = cross entropy for mutation classification and cox loss function for survival model. Models were written in python under the framework of pytorch.

### Mutation classification model

For training of the classification model, the dataset was split into four folds at the patient level for cross validation. The models were trained using cross entropy as loss function, only the top 100 tiles with the greatest scores for each of the classes were used in the backpropagation. Per-slide predictions were calculated using the average prediction of all tiles. The model performance was evaluated using Area Under the Curve (AUC) for each of the classes in a one versus the rest fashion. The final model performance was reported by the mean predicted AUC in the hold out fold. 95% CIs for the average AUC across folds were estimated using the cvAUC R package^36^. For each of the four folds, a *p* value was calculated by evaluating model predictions on the held-back fifth using Wilcoxon’s rank-sum test^37^. The resulting five *p-*values from each test were combined into a single *p* value statistic using Fisher’s method^38^. Precision-recall curves were also generated for all models for model evaluation. *Prognostic model*. In order to compare the performance of DL models to the existing evaluation system, we built deep learning (DL) models with images alone and multimodal models (Deep Miettinen) including images, tumor location and tumor size. For the Deep Miettinen model, the clinical variables were concatenated directly to the image features at the input layer (2050 features per tile including 2048 images features, tumor location as binary variable(stomach = 1, non-stomach = 0) and tumor size as continuous variable). For training of the prognostic model, the dataset was split into four folds at the patient level for cross validation. The cross validation was repeated 5 times for all models. The models were trained using cox loss function as loss function and only the top 100 tiles with the greatest scores were used in the backpropagation. Per-slide predictions were calculated using the average prediction of all tiles. The final model performance for prognosis was reported by average C-index and CI by standard deviation from cross-validation. Patients were assigned into high and low risk groups by using the mean + 0.2*standard deviation of the estimated risk scores from the training set as threshold (high risk group: risk > mean+0.2*std).

### Baseline model

To compare the predictive accuracy of DL with conventional histopathological evaluation methods, we built linear models to predict mutation classes from tumor cell types and cox models to predict prognostic risk from Miettinen risk criteria (mitotic count, tumor location and tumor size). Predicted accuracy (AUC for classification and C-index for prognosis) and *p-*values were calculated as described above using the same fold splits and repeats. All models were trained on a Tesla P40 (24 Go memory) in center 1 and tested on a NVIDIA GeForce GTX 1080 Ti (12 Go memory) in center 2.

### Expert blinded assessment of mitotic counts

We collected 824 GIST that were sent to Pathology Departments of Bergonie Institute and Centre Leon Berard for a second opinion or a systematic review in the context of the French Pathology Network for soft tissue tumors^39^ from January 2016 to December 2020. For 559 cases, mitotic count was known for both outside and reference center laboratories. The mitotic counts were categorized into two groups (more than 5 mitosis v.s less than or equals to 5 mitosis).

### Model interpretability

In order to better understand the features associated with recurrence risk and different types of mutation, a set of representative tiles were selected for pathologist examination in each condition. For high risk of recurrence, 25 tiles with the highest predicted risk scores were selected from 30 patients relapsed within 300 days. For low risk of recurrence, 25 tiles with the lowest predicted risk scores were selected from 30 predicted patients that did not relapse within 900 days. For each of the mutations, 25 tiles with the highest predicted score for the corresponding mutation from 10 positive patients were selected. All tiles were selected from the non-blurred tumor region using our tumor segmentation model and an additional blur filtering. Representatives tiles from each category were included in the main figures. These 2750 selected tiles from 110 patients were presented to 2 expert soft-tissue pathologists (RP and JMC) without the corresponding labels. The following predefined parameters were evaluated: 1- Tumor cellularity: low (<10%), medium (<10% and >50%), high (>50%), 2- Stroma if present, specify the type of stroma: fibrosis, myxoid, fibromyxoid, osseous,vacuolized pattern, 3- Cell type if single type > 50% of tumor cells, specify: spindle, pleomorphic, round, or epithelioid, otherwise: mixt, 4- Cell vacuolization: presence or absence, 5- Nuclear atypia: mild, moderate, important, 6- Hyperchromasia: presence or absence, 7- Mitosis: presence (specify number) or absence, 8- Necrosis: presence or absence, 9- Red blood cells: presence or absence, 10- Tumor infiltration if present : lymphocytes, neutrophils, other inflammatory cells, 11- Vessels: presence or absence. Statistical tests were performed in high vs low risk groups and in One v.s. the rest fashion for mutations. Two-sided T-test was used for quantitative annotations and chi-square test was performed for qualitative annotations.

### Reporting summary

Further information on research design is available in the Nature Research Reporting Summary linked to this article.

## Supplementary information

REPORTING SUMMARY

Supplementary Information

Supplementary Data 1

Supplementary Data 2

Supplementary Data 3

## Data Availability

The datasets that support the findings of this study are not publicly available due to information that could compromise research participant consent. According to French/European regulations, any reuse of the data must be approved by the ethics committee “CPP du Sud-Ouest et d’ Outre-Mer III”, Bordeaux, France. Individual participant data that underlie the results reported in this article can be shared upon request to the corresponding author.

## Code availability

Code and model weights for both mutation classification and prognostic prediction are available at https://github.com/yufuowkin/GIST.
