# Supplementary material for: Deep learning predicts patients outcome and mutations from digitized histology slides in gastrointestinal stromal tumor
Source: NPJ Precis Oncol. 2023 Jul 24;7:71. doi: 10.1038/s41698-023-00421-9 (PMC10366108; doi:10.1038/s41698-023-00421-9)
Supplement: Supplementary file 1 — REPORTING SUMMARY [file 41698_2023_421_MOESM1_ESM.pdf]

nature portfolio

Corresponding author(s): yu fu

Last updated by author(s): Dec 1, 2022

Reporting Summary

Nature Portfolio wishes to improve the reproducibility of the work that we publish. This form provides structure for consistency and transparency in reporting. For further information on Nature Portfolio policies, see our Editorial Policies and the Editorial Policy Checklist.

Statistics

For all statistical analyses, confirm that the following items are present in the figure legend, table legend, main text, or Methods section.

n/a	Confirmed	
☐	☒	The exact sample size (*n*) for each experimental group/condition, given as a discrete number and unit of measurement
☐	☒	A statement on whether measurements were taken from distinct samples or whether the same sample was measured repeatedly
☐	☒	The statistical test(s) used AND whether they are one- or two-sided<br>*Only common tests should be described solely by name; describe more complex techniques in the Methods section.*
☐	☒	A description of all covariates tested
☒	☐	A description of any assumptions or corrections, such as tests of normality and adjustment for multiple comparisons
☐	☒	A full description of the statistical parameters including central tendency (e.g. means) or other basic estimates (e.g. regression coefficient) AND variation (e.g. standard deviation) or associated estimates of uncertainty (e.g. confidence intervals)
☐	☒	For null hypothesis testing, the test statistic (e.g. *F*, *t*, *r*) with confidence intervals, effect sizes, degrees of freedom and *P* value noted<br>*Give P values as exact values whenever suitable.*
☒	☐	For Bayesian analysis, information on the choice of priors and Markov chain Monte Carlo settings
☒	☐	For hierarchical and complex designs, identification of the appropriate level for tests and full reporting of outcomes
☒	☐	Estimates of effect sizes (e.g. Cohen's *d*, Pearson's *r*), indicating how they were calculated

Our web collection on statistics for biologists contains articles on many of the points above.

Software and code

Policy information about availability of computer code

Data collection	*Provide a description of all commercial, open source and custom code used to collect the data in this study, specifying the version used OR state that no software was used.*
Data analysis	*Provide a description of all commercial, open source and custom code used to analyse the data in this study, specifying the version used OR state that no software was used.*

For manuscripts utilizing custom algorithms or software that are central to the research but not yet described in published literature, software must be made available to editors and reviewers. We strongly encourage code deposition in a community repository (e.g. GitHub). See the Nature Portfolio guidelines for submitting code & software for further information.

Data

Policy information about availability of data

All manuscripts must include a data availability statement. This statement should provide the following information, where applicable:

- Accession codes, unique identifiers, or web links for publicly available datasets
- A description of any restrictions on data availability
- For clinical datasets or third party data, please ensure that the statement adheres to our policy

All WSIs, mutations and survival data from center 1 and center 2 are available under controlled access at the European Genome-phenome Archive via accession number X (process is ongoing, will be completed at publication).

Human research participants

Policy information about studies involving human research participants and Sex and Gender in Research.

Reporting on sex and gender	The gender distribution was reported in Table 1 and table 2 for the 2 centers.
Population characteristics	Detailed patients characteristics were reported in Description of patient cohorts under methods and Table 1, 2
Recruitment	Two cohorts of patients with GIST were extracted from the Sarcoma Clinical and Biological database (https://sarcomabcb.org/), a data warehouse of clinical, pathological and molecular data.
Ethics oversight	All cases are recorded in the database of the French Sarcoma BCB (https://sarcomabcb.org/), which is approved by the National Committee for Protection of Personal Data (Commission Nationale de l'Informatique et des Libertés - CNIL, no. 910390). The study was declared to the CNIL on the 3rd April 2020 under the Sar-IA-Path project number MR 0012030420 and informed consent was obtained from all patients. The study was approved by the Institutional Review Board of both centers.

Note that full information on the approval of the study protocol must also be provided in the manuscript.

Field-specific reporting

Please select the one below that is the best fit for your research. If you are not sure, read the appropriate sections before making your selection.

☒ Life sciences ☐ Behavioural & social sciences ☐ Ecological, evolutionary & environmental sciences

For a reference copy of the document with all sections, see nature.com/documents/nr-reporting-summary-flat.pdf

Life sciences study design

All studies must disclose on these points even when the disclosure is negative.

Sample size	1233 patients from center 1 and 286 patients from center 2 were inculcated in this study.
Data exclusions	All patients from both centers were included in the mutation classification analysis. 928 patients from center 1 were excluded in the survival analysis due to missing clinical followup.
Replication	The results from the mutation classification models were replicated in the independent cohort with comparable AUC. There was an important drop of performance for the survival model due to small training set.
Randomization	For training of the classification model, the dataset was randomly split into 4 folds at the patient level for cross validation.
Blinding	NA

Reporting for specific materials, systems and methods

We require information from authors about some types of materials, experimental systems and methods used in many studies. Here, indicate whether each material, system or method listed is relevant to your study. If you are not sure if a list item applies to your research, read the appropriate section before selecting a response.

Materials & experimental systems

n/a	Involved in the study	
☒	☐	Antibodies
☒	☐	Eukaryotic cell lines
☒	☐	Palaeontology and archaeology
☒	☐	Animals and other organisms
☐	☒	Clinical data
☒	☐	Dual use research of concern

Methods

n/a	Involved in the study	
☒	☐	ChIP-seq
☒	☐	Flow cytometry
☒	☐	MRI-based neuroimaging

Clinical data

Policy information about clinical studies

All manuscripts should comply with the ICMJE guidelines for publication of clinical research and a completed CONSORT checklist must be included with all submissions.

Clinical trial registration	NA
Study protocol	NA
Data collection	Two cohorts of patients with GIST were extracted from the Sarcoma Clinical and Biological database (https://sarcomabcb.org/), a data warehouse of clinical, pathological and molecular data. All cases are recorded in the database of the French Sarcoma BCB (https://sarcomabcb.org/), which is approved by the National Committee for Protection of Personal Data (Commission Nationale de l'Informatique et des Libertés - CNIL, no. 910390). The study was declared to the CNIL on the 3rd April 2020 under the Sar-IA-Path project number MR 0012030420 and informed consent was obtained from all patients. The study was approved by the Institutional Review Board of both centers.
Outcomes	Overall survival(OS) and relapse free survival (RFS)
