# Supplementary material for: Deep learning predicts patients outcome and mutations from digitized histology slides in gastrointestinal stromal tumor
Source: NPJ Precis Oncol. 2023 Jul 24;7:71. doi: 10.1038/s41698-023-00421-9 (PMC10366108; doi:10.1038/s41698-023-00421-9)

Supplementary Figure 1. Schema of patients' treatment pathway in center 1 and center

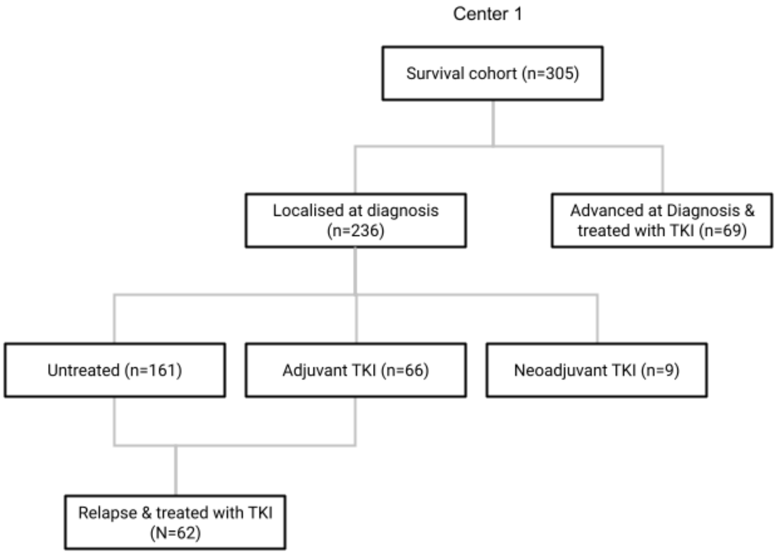

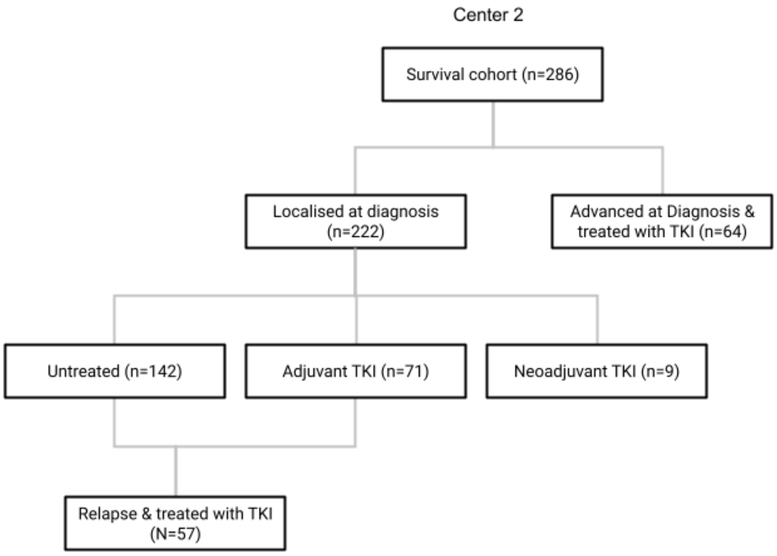

Supplementary Figure 2. Independent testing of Deep Miettinen model for localized, untreated GIST in center 2. Kaplan–Meier plots shown for RFS for localized untreated patients within different categories. From left to right: risk groups defined by Deep Miettinen; risk groups defined by Miettinen(cutoff for high risk was re-estimated so that the number of

high risk patients is comparable to the number defined by Miettinen evaluation system high risk patients); risk groups defined by deep Miettinen for intermediate risk patients defined by Miettinen; risk groups defined by deep Miettinen for high risk patients defined by Miettinen.

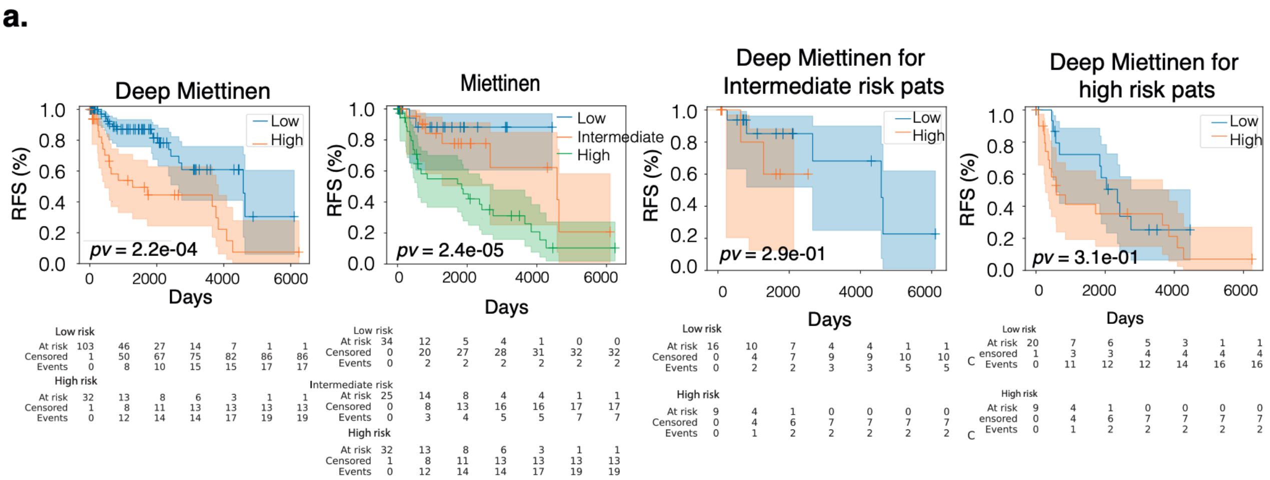

Supplementary Figure 3. Precision-Recall curves for mutation classification. a. mutation classification at gene level. Shown for all samples (left) and samples from stomach tumors only (right). **b.** mutation classification at exon level. **c.** mutation classification at codon level.

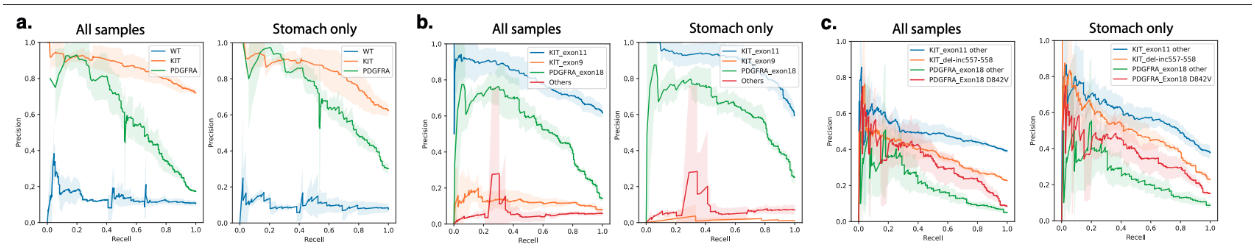

Supplementary Figure 4. Comparison of performance in mutation classification between DL on HES slides and tumor cell types. a. Barplot with AUC values for mutation classification at gene level. Shown for all samples (left) and samples from stomach tumors

only (right). Each bar corresponds to the average AUC from 4-fold cross validation. AUCs of DL models are shown in blue and AUCs of models based on tumor cell types are shown in orange. Error bars indicate the CI estimated using cvAUC. **b.** Barplot with AUC values for mutation classification at exon level. **c.** Barplot with AUC values for mutation classification at codon level. **a-c** from center 1 and **d-f** from center2.

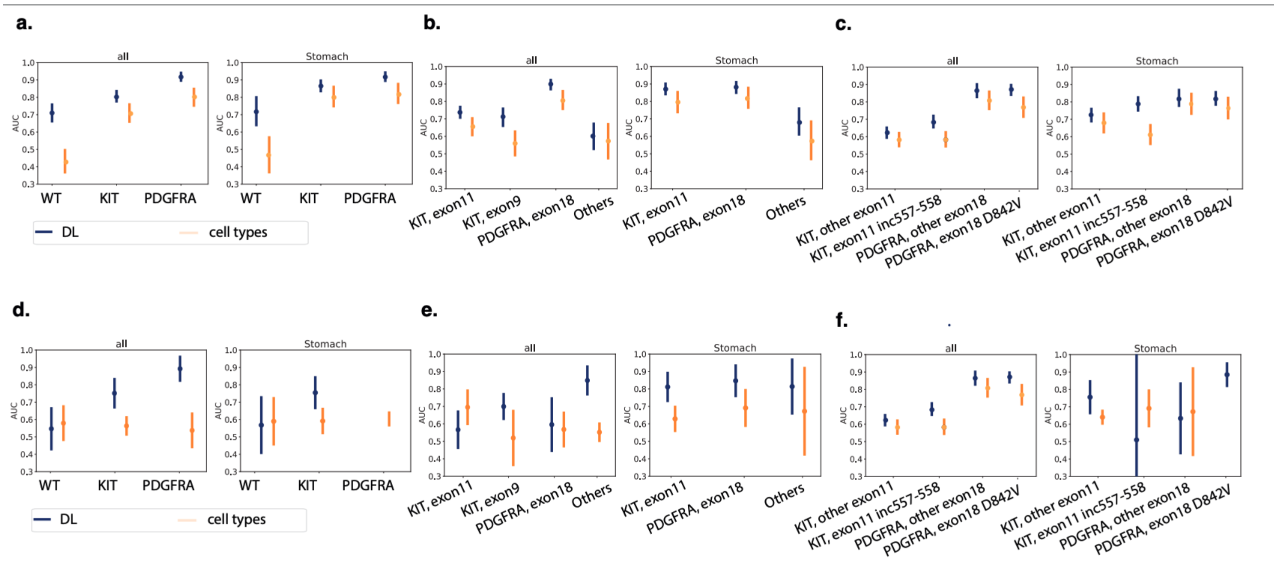

Supplement: Supplementary file 2 — Supplementary Information [file 41698_2023_421_MOESM2_ESM.pdf]
